# Monomeric C‐reactive protein: A novel biomarker predicting neurodegenerative disease and vascular dysfunction

**DOI:** 10.1111/bpa.13164

**Published:** 2023-05-09

**Authors:** Ylenia Pastorello, Roxana O. Carare, Claudia Banescu, Lawrence Potempa, Mario Di Napoli, Mark Slevin

**Affiliations:** ^1^ Department of Anatomy George Emil Palade University of Medicine, Pharmacy, Science and Technology Târgu Mures Romania; ^2^ Clinical and experimental Sciences University of Southampton Southampton UK; ^3^ Department of Life Sciences, College of Science, Health and Pharmacy Roosevelt University Schaumburg Illinois USA; ^4^ Department of Neurology and Stroke Unit San Camillo de Lellis General Hospital Rieti Italy; ^5^ Manchester Metropolitan University Manchester UK

**Keywords:** monomeric C‐reactive protein, neurodegeneration, vascular dysfunction

## Abstract

Circulating C‐reactive protein (pCRP) concentrations rise dramatically during both acute (e.g., following stroke) or chronic infection and disease (e.g., autoimmune conditions such as lupus), providing complement fixation through C1q protein binding. It is now known, that on exposure to the membranes of activated immune cells (and microvesicles and platelets), or damaged/dysfunctional tissue, it undergoes lysophosphocholine (LPC)‐phospholipase‐C‐dependent dissociation to the monomeric form (mCRP), concomitantly becoming biologically active. We review histological, immunohistochemical, and morphological/topological studies of post‐mortem brain tissue from individuals with neuroinflammatory disease, showing that mCRP becomes stably distributed within the parenchyma, and resident in the arterial intima and lumen, being “released” from damaged, hemorrhagic vessels into the extracellular matrix. The possible de novo synthesis via neurons, endothelial cells, and glia is also considered. In vitro, in vivo*,* and human tissue co‐localization analyses have linked mCRP to neurovascular dysfunction, vascular activation resulting in increased permeability, and leakage, compromise of blood brain barrier function, buildup of toxic proteins including tau and beta amyloid (Aβ), association with and capacity to “manufacture” Aβ‐mCRP‐hybrid plaques, and, greater susceptibility to neurodegeneration and dementia. Recently, several studies linked chronic CRP/mCRP systemic expression in autoimmune disease with increased risk of dementia and the mechanisms through which this occurs are investigated here. The neurovascular unit mediates correct intramural periarterial drainage, evidence is provided here that suggests a critical impact of mCRP on neurovascular elements that could suggest its participation in the earliest stages of dysfunction and conclude that further investigation is warranted. We discuss future therapeutic options aimed at inhibiting the pCRP‐LPC mediated dissociation associated with brain pathology, for example, compound 1,6‐bis‐PC, injected intravenously, prevented mCRP deposition and associated damage, after temporary left anterior descending artery ligation and myocardial infarction in a rat model.

## Graphical abstract

The extraordinary complexity, and pathological biological activity, of monomeric C‐reactive protein within the brain parenchyma.

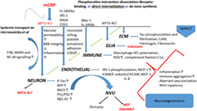

## 1 CRP BACKGROUND

C‐reactive protein (CRP) is a homo‐pentameric acute phase protein, first discovered by Tillett and Francis in 1930, and characterized by its ability to precipitate the “C” polysaccharide derived from the pneumococcus cell wall. Transcriptional stimulation of the CRP gene chromosome 1 takes place in the hepatocytes, especially in relation to raised levels of circulating interleukin‐6 (IL‐6), which is in fact the main inducer of CRP gene expression. CRP secretion is furthermore enhanced by other cytokines, for example, Interleukin 1 (IL‐1). Through binding with C1q, CRP is able to activate the classical complement pathway which leads to promotion of phagocytosis and pathogen clearance. Moreover, CRP reacts with cells at the sites of tissue injury and, after binding to phosphocholine (PC), phospholipids, histone, chromatin, and fibronectin, it plays a pivotal role in recognition and removal of damaged cells. Native pentameric CRP (nCRP) displays a half‐life of 19 h in plasma, both in physiological or pathological environments; its levels augment and decrease promptly with the onset and elimination of the inflammatory stimulus, respectively. CRP is recognized and extensively used as a marker of disease activity. Standard CRP testing in plasma represents the investigation of choice in clinical practice for detection of suspected inflammation, in particular to assess lower CRP levels (0.3–1.0 mg/L). High sensitivity CRP (hs‐CRP) testing is advisable as it offers additional information regarding cardiovascular risk and its stratification [1, 2]. Elevation of CRP baseline concentrations mirrors inflammatory responses in the acute setting; it is indeed broadly acknowledged that increased CRP levels are encountered in patients with appendicitis, pancreatitis, cholecystitis, meningitis and, interestingly, in hemorrhagic stroke. Recently, a fascinating hypothesis which attributes a clinical significance of CRP in chronic inflammatory pathologies, and specifically in neurodegenerative diseases, has been formulated. Namely, CRP represents an inflammatory biomarker and risk predictor in insulin resistance, progressive visual impairment, age‐related macular degeneration, neurodegenerative disorders with associated motor symptoms, and autoimmune disorders like rheumatoid arthritis (RA) and inflammatory bowel disease (IBD). A substantial influence on cardiovascular disease pathogenesis seems to be exerted by the more biologically active subunit of CRP, that is, monomeric CRP (mCRP) [3, 4].

## 2 MCRP AND ITS BIOLOGICAL ACTIVITY

Pentameric CRP irreversibly dissociates into five subunits at the site of infection and inflammation. Dissociation into free monomers has been reported to take place either at elevated temperatures in the absence of calcium, or in the presence of high levels of urea [1, 5]. Conversion to mCRP leads inevitably to a prompt mutation of the proinflammatory profile of the protein; and it is in fact mCRP, which is the culprit of the inflammatory pathogenesis as a result of its perpetual interaction with endothelial cells (ECs), neutrophils, macrophages, and platelets. Specifically, conformational changes of nCRP to mCRP occur due to the binding of CRP subunits to PC residues of lysophosphatidylcholine (LPC) localized on cell membranes, followed by interaction with specific receptors at membrane lipid‐rafts. Generation of LPC is a consequence of the action of Phospholipase A2 (PLA2) enzymes on cell surface lysophospholipids. Interaction with the PC localized on activated platelets and LPC residues on oxidized low‐density lipoprotein (ox‐LDL), by lipoprotein‐associated PLA2 (LP‐PLA2), circumscribes mCRP production to the inflammatory micro‐environments such as atherosclerotic plaques. mCRP is furthermore held responsible for the upregulation of monocyte chemoattractant protein‐1 (MCP‐1), intercellular adhesion molecule 1 (ICAM‐1), vascular cell adhesion molecule 1 (VCAM‐1), endothelial leukocyte adhesion molecule‐1 (ELAM‐1), and interleukin 8 (IL‐8) in EC, directly leading to adhesion of neutrophils, with the p38 MAPK signaling pathway being the mediator of this cascade of events. The ability of mCRP to promote monocyte recruitment and their adherence to endothelium, activation of the inflammatory macrophage phenotype (M1) and their uptake of ox‐LDL, with ensuing formation of foam cells are also considered to be key pathological mechanisms in peripheral vascular and cardiovascular diseases [6, 7]. Implications of mCRP biological activity are reflected in its disease modifying behavior, especially in the vascular components of neurodegenerative and cardiovascular pathologies as this review aims to emphasize.

## 3 MCRP IN NEUROINFLAMMATION AND NEURODEGENERATIVE DISEASES

Neuroinflammation displays a plethora of origins. Both acute and chronic inflammatory conditions, through an extended heterogeneity of mechanisms, can generate and subsequently influence the course of this central nervous system (CNS) inflammatory response. Acute traumatic brain injury (TBI), ischemic or hemorrhagic stroke, and serious infections such as meningitis are always accompanied by a degree of neuroinflammation. In the chronic setting, lower grade but equally potentially harmful etiology emanating from a variety of systemic conditions such as autoimmune disease, including giant cell arteritis (GCA), multiple sclerosis (MS) and IBD, as well as other long‐standing pathologies, comprising type II diabetes mellitus (T2DM), osteoarthritis (OA), periodontal disease et al., account as causes of neuroinflammatory initiation and progression [4, 8].

Neuroinflammation entails four conventional mechanisms, as eloquently synthesized by Moyse et al., namely:

- Elevated tissue concentration of pro‐inflammatory cytokines, matrix metalloproteinases, prostaglandins, and reactive oxygen and nitrogen species (ROS and RNS, respectively);
- Microgliosis and astrocytosis and, if the homeostatic balance is continuous, microglial priming;
- Dysfunction within the neurovascular units (NVUs), permeabilization of the blood brain barrier (BBB), and consequent penetration of active monocytes, macrophages, and T‐lymphocytes;
- Development of a highly toxic micro‐environment followed by compromised neuronal survival and eventual death [8, 9, 10].

Elevated baseline levels of CRP in the chronic inflammatory milieu raise questions regarding its probable impact on neuroinflammation, as both causative factor and perpetuating insult. Hsuchou et al. [11] demonstrated in mice how high CRP concentrations are able to increase the BBB paracellular permeability, therefore allowing its own entry into the CNS, with further induction of reactive gliosis and instigation of dysfunction. This pathological interaction requires further in‐depth study.

**FIGURE 1:**
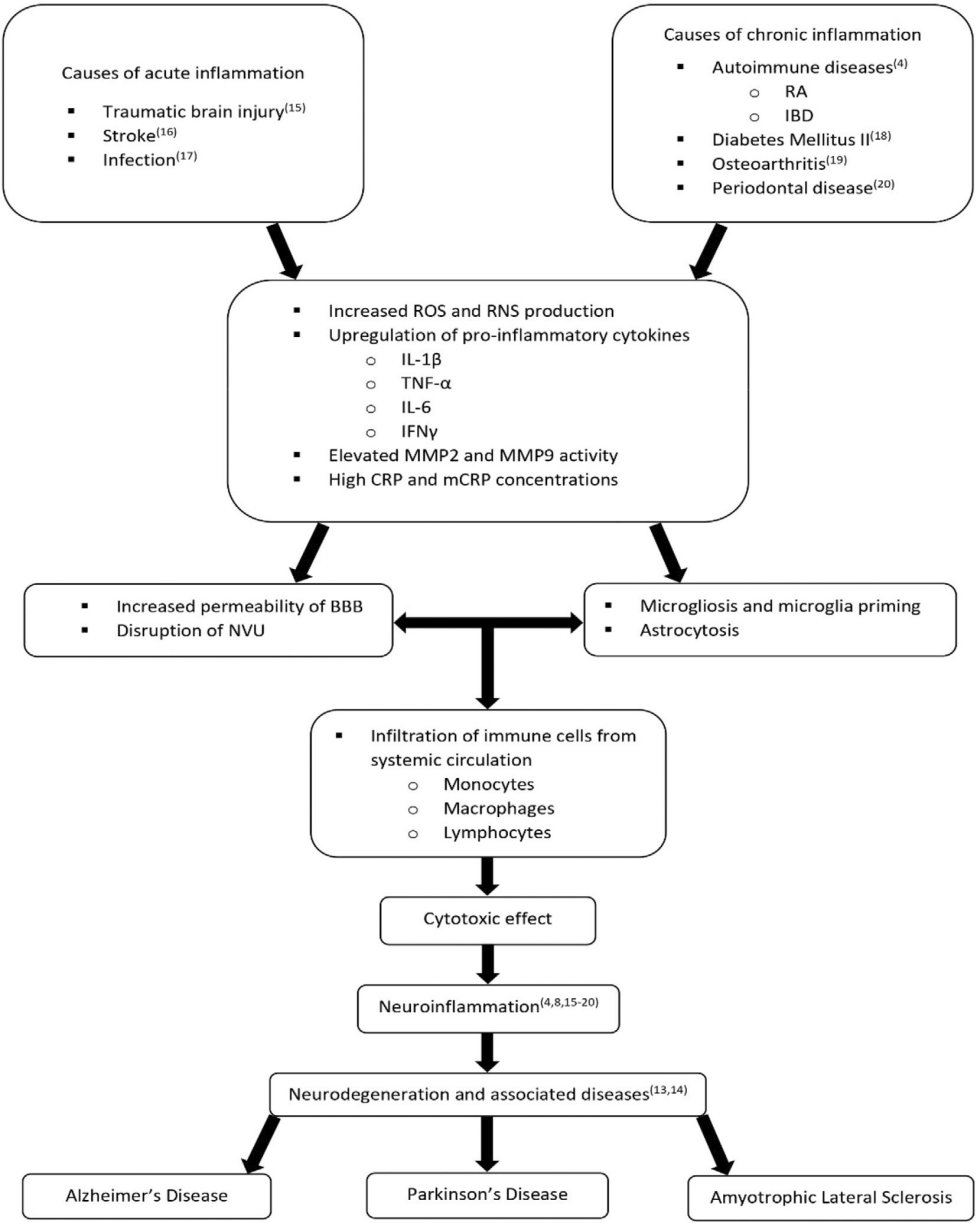
Inflammatory mechanisms leading to neuroinflammation and subsequent neurodegenerative diseases development. BBB, blood brain barrier; CRP, C‐reactive protein; IBD, inflammatory bowel disease; IFNγ, interferon gamma; IL‐1β, interleukin 1 beta; IL‐6, interleukin 6; mCRP, monomeric C‐reactive protein; MMP2, matrix metalloproteinase 2; MMP9, matrix metalloproteinase 9; NVU, neurovascular unit; RA, rheumatoid arthritis; RNS, reactive nitrogen species; ROS, reactive oxygen species; TNF‐α, tumor necrosis factor alpha [4, 8, 15, 16, 17, 18, 19, 20].

Similarly, the monomeric form of CRP has been shown to exert both morphological and functional negative effects: Slevin et al. [12] exhibited indeed how, in the presence of mCRP, inter‐cellular gap dimensions were remarkably broader and vascular monolayer permeability was augmented in rodent models. Hence, neuroinflammation appears to be a mosaic composed of triggering events and elements, all playing key roles in what has been widely acknowledged as pathogenesis of neurodegenerative diseases, for example, Alzheimer's disease (AD), Parkinson's disease (PD), and amyotrophic lateral sclerosis (ALS) (Figure 1) [13, 14].

## 4 MONOMERIC CRP DYNAMICS WITHIN THE BRAIN AND EFFECTS UPON BLOOD VESSEL PHYSIOLOGY AND VASCULARIZATION

During the process of inflammation, CRP, as already described above, dissociates on activated platelets and macrophages and this travels primarily within the systemic circulation and homes to or “stick” to sites of injury where it may then contribute to perpetuation of inflammation and disease progression and acute events such as thrombosis. However, mCRP also becomes associated with circulating microparticles (MPs) and cell‐derived exosomes originating from activated cell membranes, which are increased significantly during inflammatory states and have been shown to be associated with increased arterial damage, atherosclerosis, and myocardial infarction (MI) [21, 22, 23].

Carriage of mCRP by these MPs seems one logical mechanism through which they can travel more easily through even an intact BBB and contribute to the potential load within the brain, entering the brain extracellular matrix (ECM) via compromised microvessels even at the level of the NVU. Previous studies have already demonstrated increased generation of plasma MPS following ischemic stroke potentially supporting the later development of dementia [24], whilst several reports have documented a strong relationship between circulating MPs and vascular risk factors of dementia [25] and AD directly [26]. Wang et al. [27] found that total circulating pCRP was inversely correlated with APOE ε4 (a critical risk factor associated with gliovascular function and risk of AD [28]); and associated amyloid beta 42 (Aβ42) in the cerebrospinal fluid (CSF).

However it is now known that total circulating CRP does not inform and is not correlated with plasma born circulating mCRP levels which now are accepted to be the important biomarker, and a novel risk factor; in this regard, Gan et al. [29] recently confirmed that mCRP directly stimulated cellular AD pathogenesis using murine cortical APOE ε4‐knock‐in neurons. From a purely inflammatory perspective, mCRP attached to microvesicles in the plasma of patients with sepsis was shown to be responsible for macrophage‐secreted IL‐8 which is strongly associated with BBB breakdown in AD [30, 31]. Since sepsis is closely associated with later cognitive impairment and AD, in children, the impact of mCRP should be given urgent attention [32, 33, 34].

**FIGURE 2:**
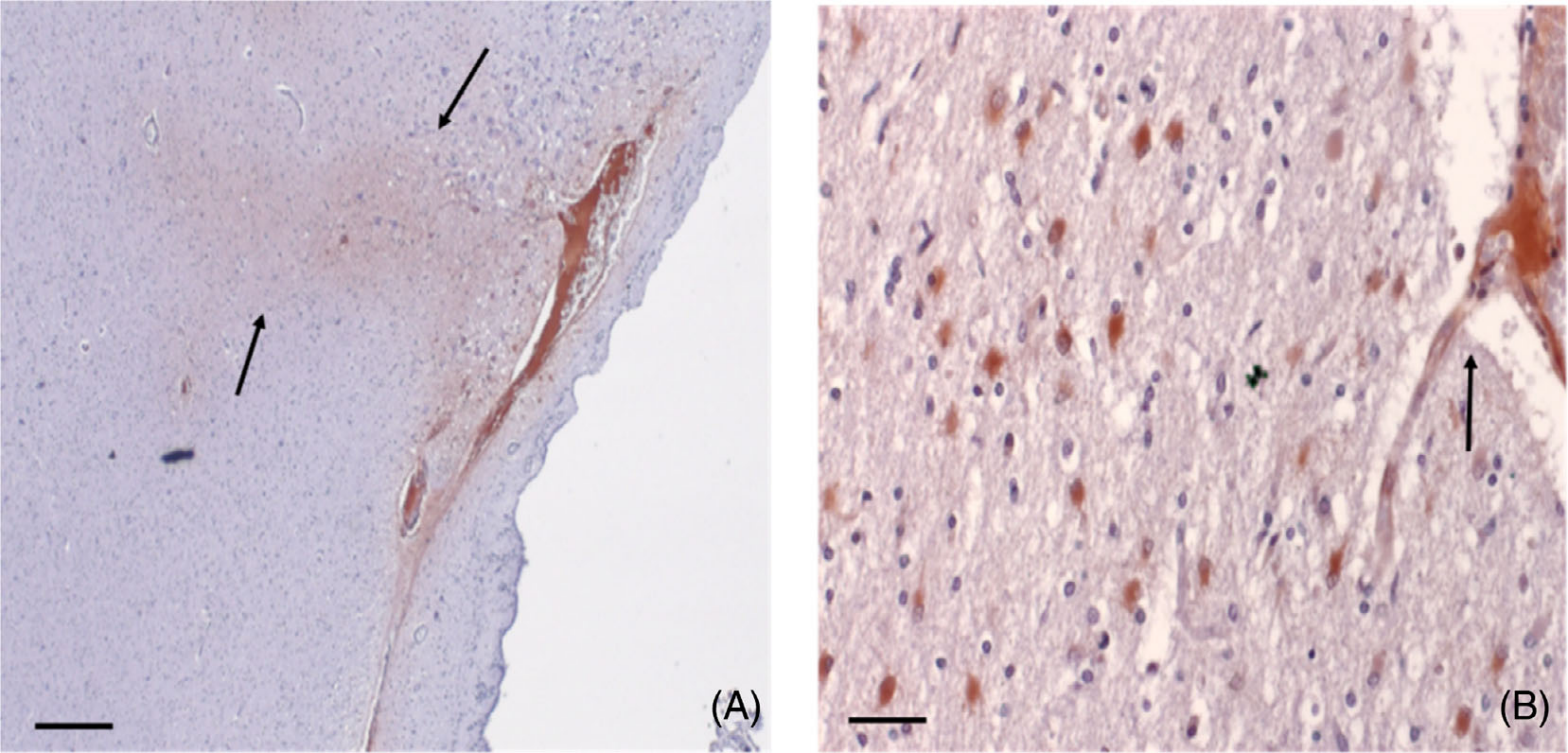
Monomeric C‐reactive protein (mCRP) is present in significant amounts in the tissue and extracellular matrix of infarcted human post‐mortem brain tissue (from the series listed in Al‐Baradie et al. [32]: Here, we show (left)‐microvascular “pockets” of mCRP (DAB brown; scale bar 200 μm) infiltrating the local cerebral parenchyma (arrows) and (right)‐magnified appearance of a leaking cortical microvessel with localized mCRP‐positive inflammatory reaction and immune‐infiltration (arrow; scale bar 100 μm; DAB brown development).

Of course, during infarction or hemorrhage, CRP from the micro‐circulation is also in direct contact with inflamed tissue and creates hotspots of mCRP emanating from the core/origin (Figure 2; [35]).

**FIGURE 3:**
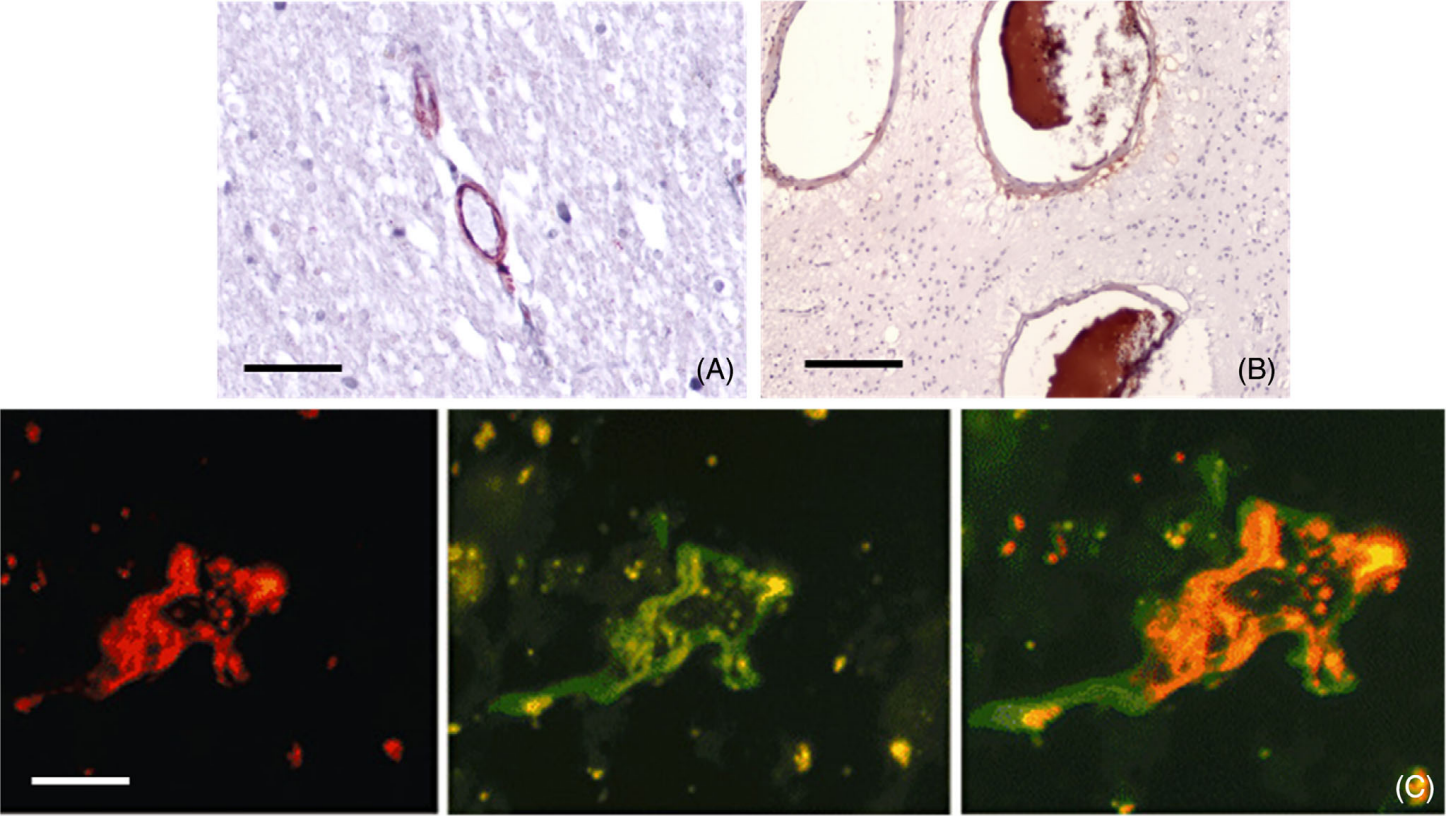
(A, B) Parenchymal microvessels from human brain cortex (Alzheimer's disease [AD] confirmed by Braak‐Tangle index (stage 4–6) and the AD‐ABC score (2+ in each category), originating from the Bristol, UK Brain Bank as shown in Reference [32], IHC stained with antibody against monomeric C‐reactive protein (mCRP) (8C10), showing luminal (apical) mCRP present in microvessels (A, double labelled to show β‐amyloid, grey), medium sized vessels in inflammatory white matter region (arterioles) (B). (C) Ischemic stroke post‐mortem grey matter cortex obtained from the Brain Bank at the Institut de Neuropatologia, Servei Anatomia Patològica, IDIBELL, Hospital Universitari de Bellvitge, Barcelona [39], shows the cross‐section of an activated microvessel. Left image‐rhodamine red CD105 and middle image FITC‐mCRP visualization and C double staining‐showing co‐localization in an active peri‐infarcted (penumbral) vessel staining; (A, B: scale bar 200 μm [C] scale bar 100 μm).

With luminal “resident” mCRP, the functional capacity and patency may become impaired over time. The apical‐luminal association of mCRP in damaged vessels of various sizes is shown in Figure 3A–C, and Li et al. [36], confirmed that mCRP induced pro‐inflammatory EC activation through this specific topological orientation, suggesting the importance of microvessel EC‐activation in mediating the neuro‐inflammatory response after brain injury. The potential role and mechanisms of pathogenic stimulation of mCRP in blood vessels was described as early as 2009 [37]. More recently, Thiele et al. [38] demonstrated that only the dissociated mCRP could induce leukocyte‐EC interaction and recruitment following rat kidney ischemia‐reperfusion injury and stereotactic injection of pCRP, and this was further confirmed in biopsied human muscle.

**FIGURE 4:**
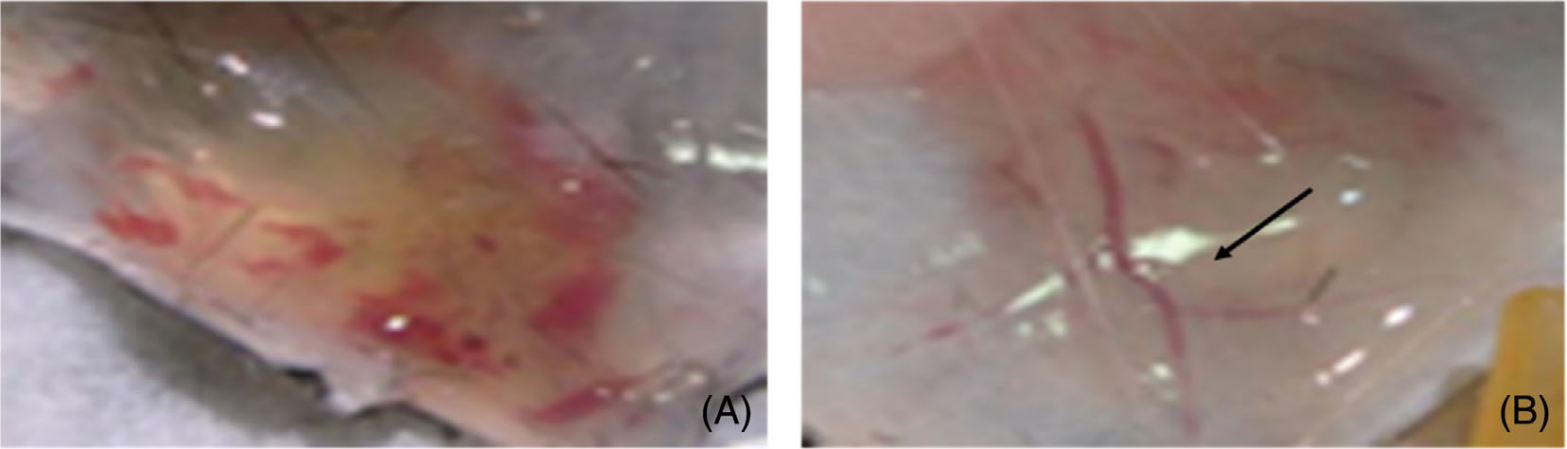
Subcutaneously implanted collagen plugs seeded with human microvessel endothelial cells (ECs) were exposed to monomeric C‐reactive protein (mCRP) in mice. (A), shows formation of an extended but hemorrhagic network of microvessels both in the plug and the overlying skin tissue, while ECs exposure to pCRP induced a much milder but stable increase in micro‐vasculature (B; arrow); (Taken from 40; with permission from the publisher).

Further investigation into the mechanisms through which mCRP stimulates EC activation and immune cell response and recruitment revealed that angiogenesis—albeit often resulting in production of immature or leaky vessels with increased permeability [12], was dependent upon F3‐gene associated tissue factor expression after treatment of microvascular EC in vitro [40]. Ullah et al. [41] found that mCRP‐induced EC‐monocyte adhesion occurred in a fibronectin‐dependent fashion both in vitro and in vivo, and this is a critical early step in the extravasation process leading to increased inflammation associated with vascular disease such as atherothrombosis (Figure 4) [42].

**FIGURE 5:**
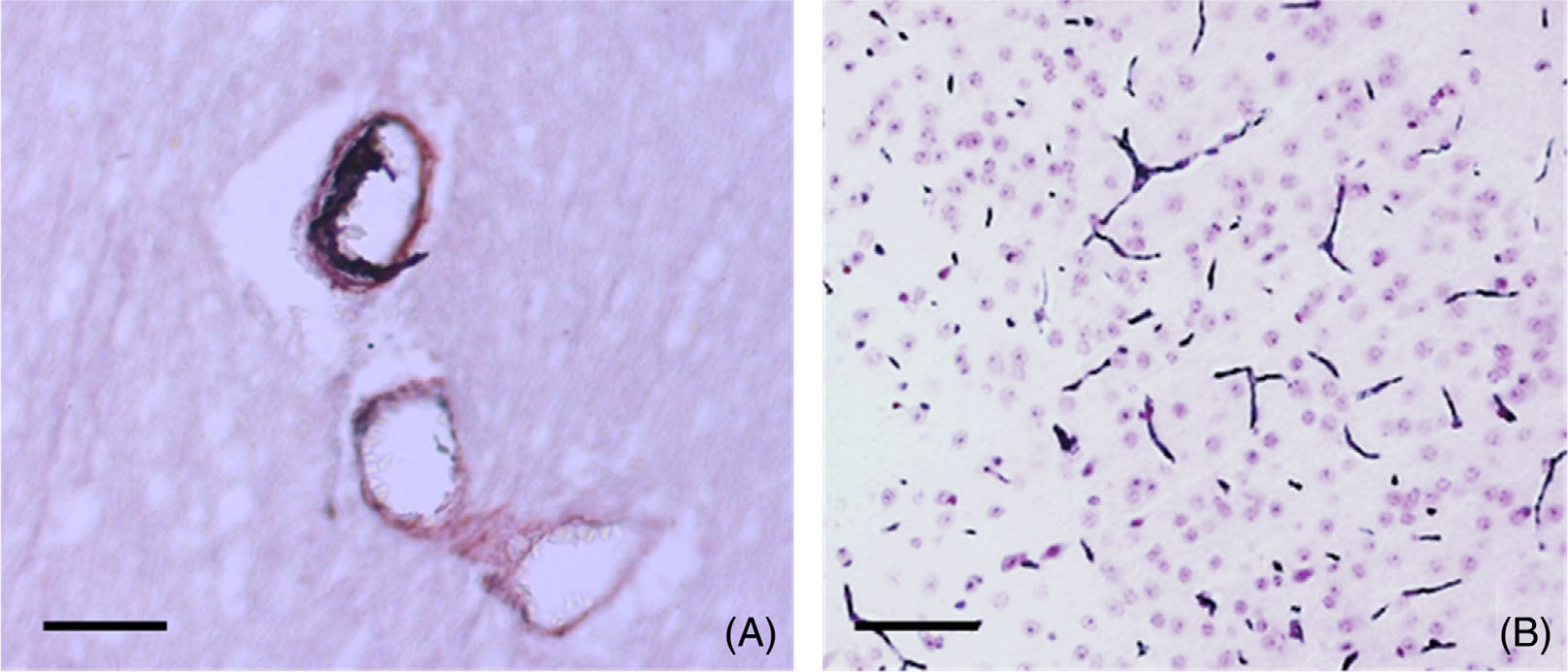
Vascular penetration and cortical microvessel loading (AD confirmed samples by Braak‐Tangle index (stage 4–6) and the AD‐ABC score (2+ in each category); originating from the Bristol, UK Brain Bank; taken from article cited as 32 and used with permission of the publisher). (A), shows post‐mortem IHC image from parietal region white matter and co‐staining of intra‐luminal monomeric C‐reactive protein (mCRP) (antibody 8C10; DAB brown) with vessel wall β‐amyloid positive co‐localization (Nova red; 32). Panel (B) shows wide cortical distribution of mCRP in microvessels 1 month after stereotactic CA1 hippocampal injection in a murine model associated (and remained up to the 6‐month end‐point of the study); with later decline in memory and motor function and neurodegenerative pathology (the vessels also stained positive for CD105 a marker of endothelial cell activation; shown here is DAB‐gray‐positive development, scale bar in [A]200 μm and [B] 100 μm).

The most recent studies have provided evidence linking vascular inflammation with neurological and cognitive decline associated with AD, and in this regard, Zhang et al. [43], found that intraperitoneal injection of mCRP into ApoE4 knock‐in mice, caused abnormal vascular development and increased T lymphocyte extravasation in a CD31‐dependent mechanism, resulting in increased cognitive deficit (Figure 5).

Taken together, evidence presented above raises the following questions: (1) What is the vascular spreading mechanism through which mCRP permeates the ECM of the brain and reaches previously naïve or “normal looking” non‐damaged regions? (2) What direct impact does mCRP have in the ECM and following cellular binding or internalization?

**FIGURE 6:**
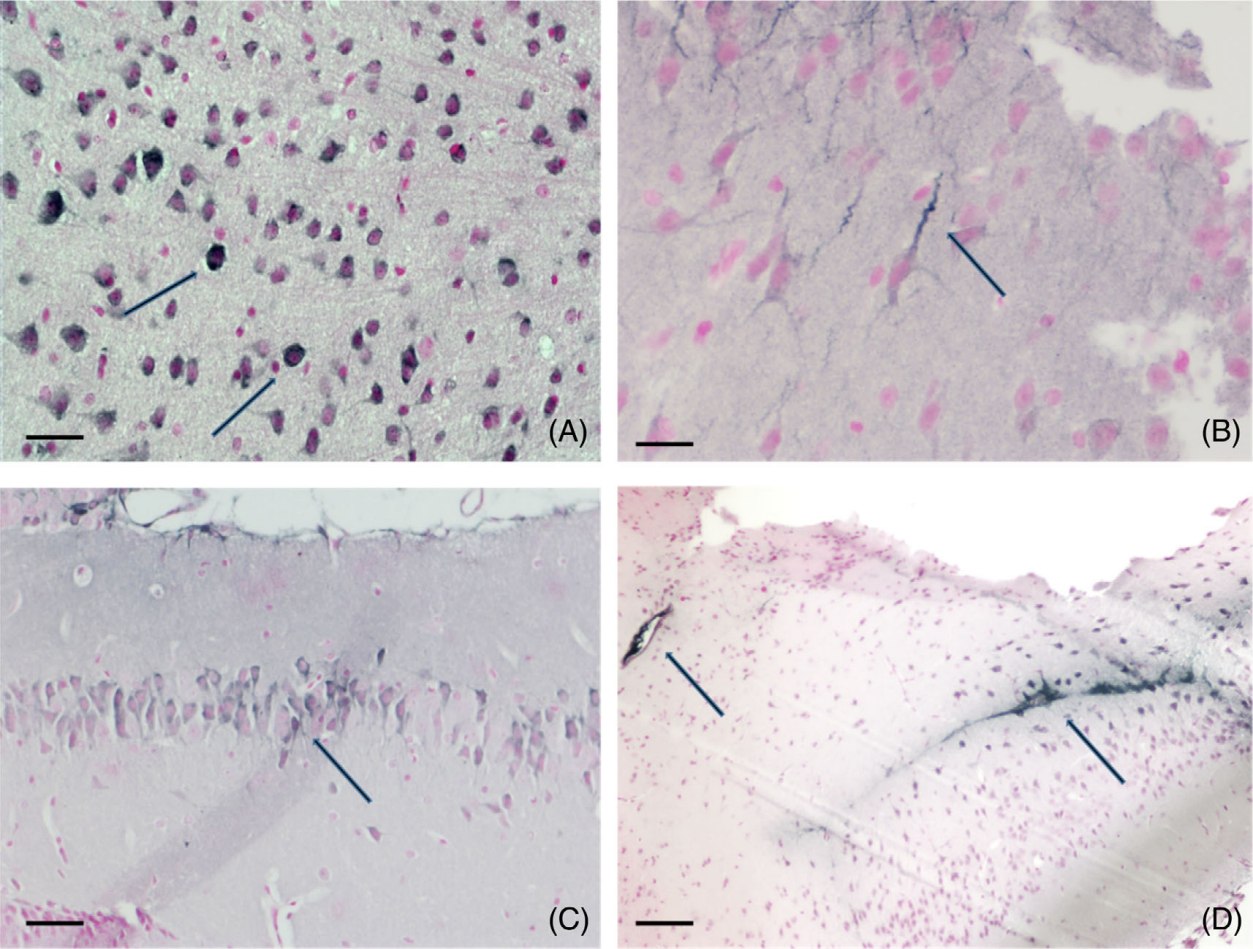
Monomeric C‐reactive protein (mCRP) was stereotactically delivered into the hippocampal CA1 region of wild‐type mice and morphological and topological localization was observed by IHC (DAB grey‐black development) after 1–6 months. Here, nuclear expression is seen in neocortical neurons (A) with positive axonal staining (A,B) and in (C), mCRP‐peri‐nuclear staining of hippocampal neurons in CA1 and (D), shows mCRP near the injection site (arrow on the right) and strong expression in an adjacent blood vessel (left arrow) and dorsal and lateral ventricles (Images taken from the panels in Slevin et al.; [12], with permission of the publisher). Scale bar in (A,B) 200 μm, (C) 100 μm, and (D) 50 μm.

Confirming the brain systemic potential effects, notable uptake of mCRP was seen in widespread cortical and hippocampal microvessels and neurons following stereotactic injection of mCRP into the CA1 region of normal mice (Figure 5, 6) [12]. It is not straight forward to understand how the protein becomes so effectively distributed throughout the brain parenchyma; it may diffuse through the extracellular spaces, it may enter the basement membranes of capillaries and arteries or there may be additional de novo synthesis through an unknown pathophysiological response (see later).

**FIGURE 7:**
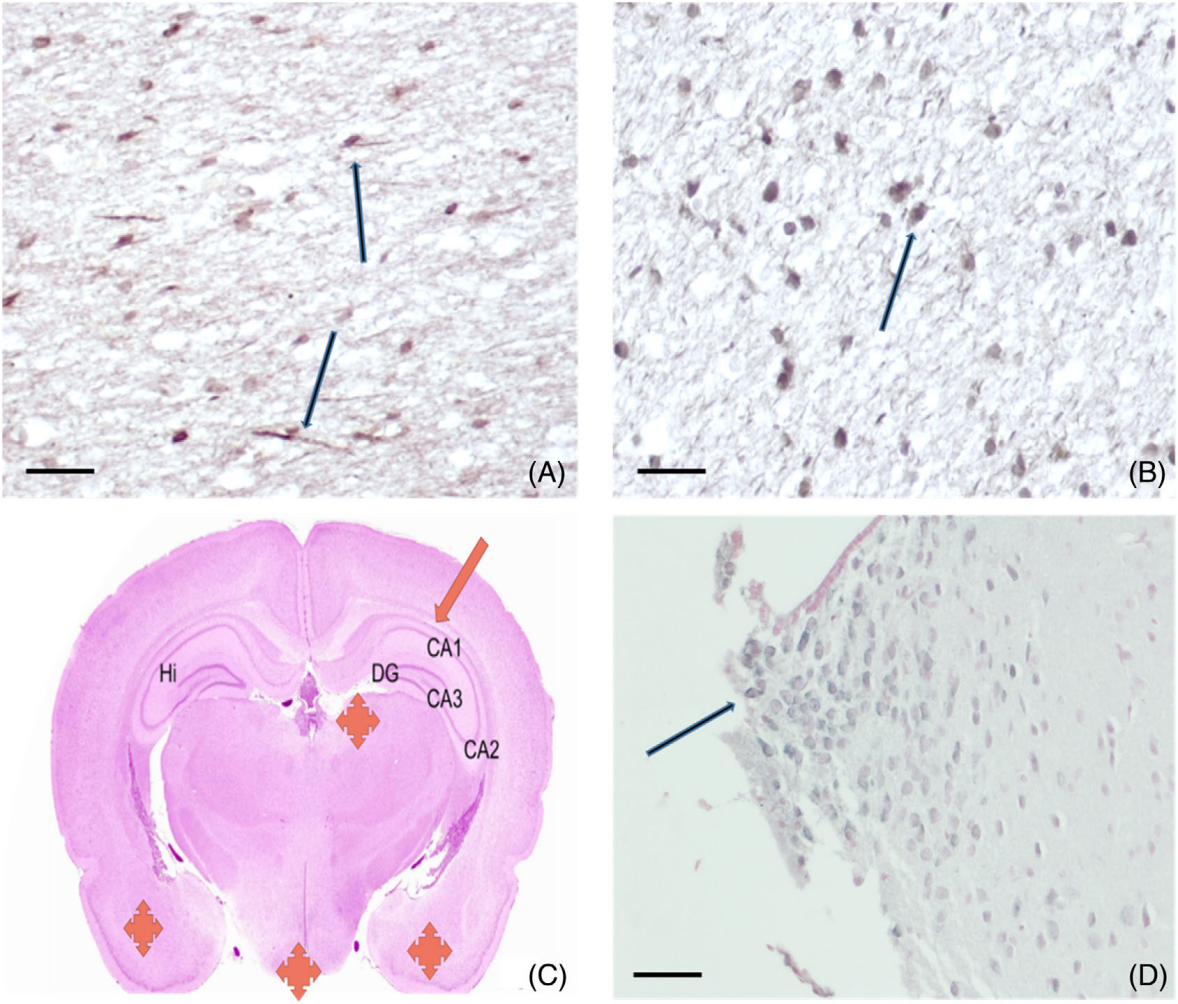
Strong staining in the hypothalamus in patients with AD (taken from Al‐Baradie et al.; [32], with permission from the publisher; (A) microvessels and microglia positive for monomeric C‐reactive protein (mCRP) (arrows) and nuclear localization in cortical neurons (B), in both cases, the hypothalamus appeared vacuolated and presented with abnormal degenerative and inflammatory morphology (DAB‐brown staining; ×100). The panel in (C), shows the CA1 localization of mCRP after stereotactic injection in the murine model (arrow), and the multi‐headed arrows show the subsequent mCRP‐positive regions within 1‐month of injection. (D), segment of hypothalamus as represented by the central multi‐headed arrow in (C); strong mCRP‐peri nuclear staining can be seen (arrow; Scale bar in A–C: 100 μm; DAB brown).

In addition, studies have shown that remote regions of the brain including the hypothalamus also become mCRP‐positive as shown by example in Figure 7, panels (C,D), with panels (A,B) showing hypothalamic‐cytoplasmic positive neurons and microglia in post‐mortem samples analyzed by IHC after hemorrhagic stroke [44, 45]. Cells with the distinctive morphological appearance of astrocytes were also positively stained throughout the peri‐infarcted zone, in post‐mortem samples from ischemic stroked regions [32], whilst and similar findings were seen in a murine model within 1‐month following stereotactic injection of mCRP into the hippocampus CA1 in wild‐type mice (Figure 7D).

**FIGURE 8:**
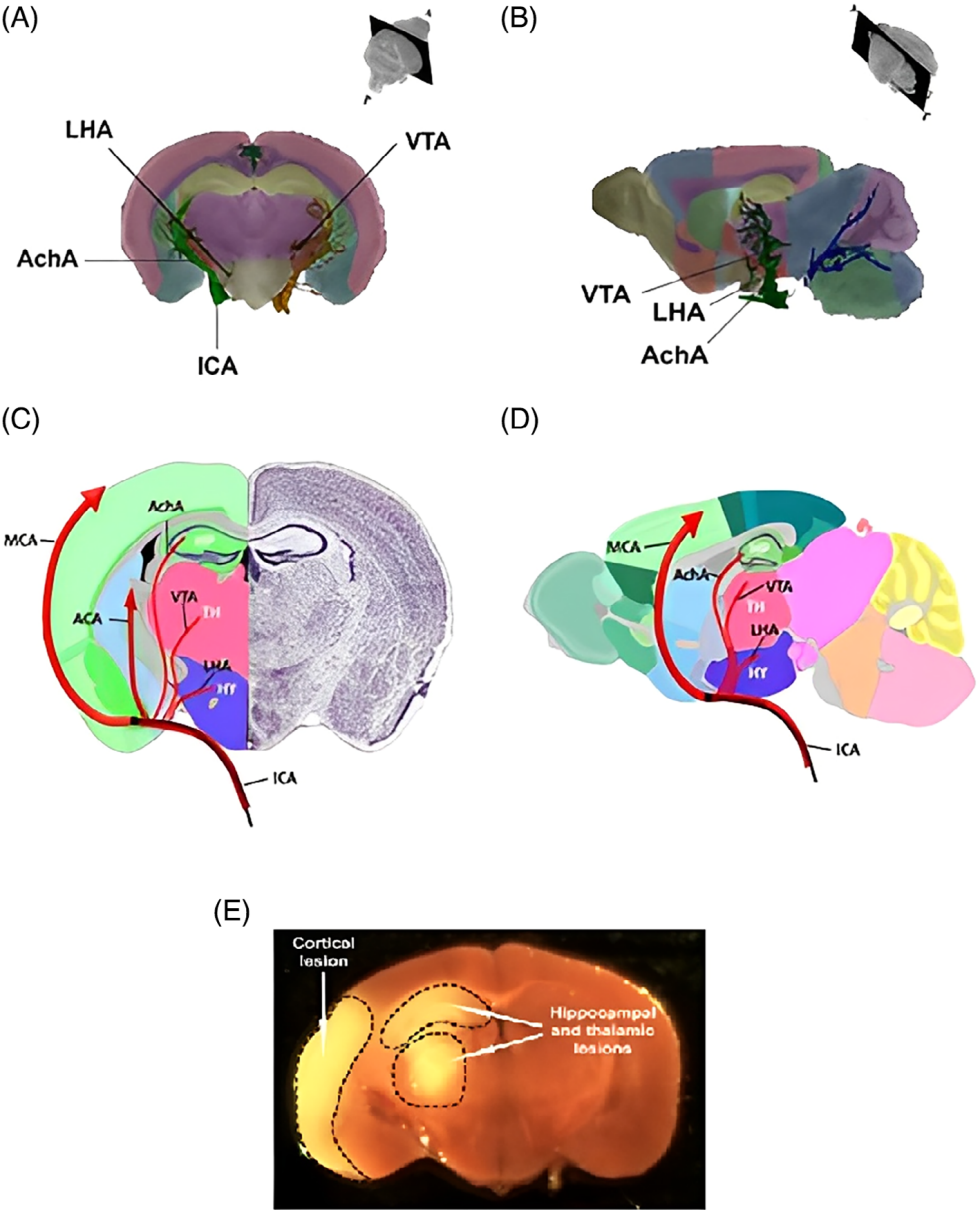
Small deep arteries and non‐specific lesions in the mouse brain after MCAO. (A) Coronal and (B) Sagittal magnetic resonance brain slices showing the ending of internal carotid artery in mouse (taken from Dorr et al.; [46], with permission of the authors and the publisher). (C,D) Simplified scheme of the small arteries supplying hippocampus, hypothalamus, and thalamus in coronal (C), and sagittal (D), a mouse brain section. Adapted from Allen Brain Atlas, the black line into internal carotid artery represents the occlusion by the filament. (E) A coronal mouse brain slice showing cortical, thalamic and hippocampal lesions after middle cerebral artery occlusion (arrows) [47]. ACA, anterior cerebral artery; AchA, anterior choroidal artery; HP, hippocampus; HY, hypothalamus; ICA, internal carotid artery; LHA, lateral hypothalamic artery; MCA, middle cerebral artery; TH, thalamus; VTA, ventral thalamic artery.

An explanation for movement of mCRP through the brain vasculature could be suggested based on the physiological and anatomical structure of the vessels supplying the brain. For example, in the mouse brain, beyond the circle of Willis, there are small and deep arteries which play a role in the blood supply of deep areas, such as the anterior choroidal artery (AchA), the lateral hypothalamic artery (LHA) and the ventral thalamic artery (VTA). This deep vascularization has been detailed in the “Mouse Cerebral Vasculature Brain Atlas” which traces vascular anatomy and morphology of brain vasculature [46]. The AchA, a minor branch of the internal choroid artery, provides the major blood supply to the piriform cortex and the amygdala and to a minor extent to the anterior portion of hippocampus. The artery then divides into other vessels supplying the thalamus, lateral ventricle, choroid plexus, and third ventricle. LHA and VTA, which can originate from the posterior communicating artery, respectively irrigate the lateral edge of the hypothalamus and the floor of the thalamus. These blood vessels can also originate directly from the internal carotid artery, proximally to the origin of MCA (Figure 8A,B). Consequently, proximal occlusion of the MCA may produce damage in the anterior hippocampus, thalamus, and/or hypothalamus (Figure 8C,D) whereas distal occlusion results solely in lesions of MCA territory of striatum, piriform cortex, and portions of the parietal–temporal cortex.

In AD, there is deposition of amyloid‐beta (Aβ) in the extracellular spaces of the brain as plaques and in the walls of cerebral arteries and capillaries (very rarely veins) as cerebral amyloid angiopathy, or CAA [48]. The deposition of Aβ in CAA occurs in the basement membranes of capillaries and arteries, effectively the intramural periarterial drainage pathways of the brain [49]. In CAA, the expression of mCRP is closely associated with the vascular Aβ as well as with the CD68 and IL1β markers of neuroinflammation [32]. It remains to be seen if mCRP is a marker of CAA or of CAAri, a subset of CAA where there are autoantibodies against Aβ in the CSF [50].

## 5 MCRP LOCALIZATION IN NEURONS AND OTHER CELLS; ASSOCIATION WITH NEURODEGENERATIVE PATHOLOGY AND POTENTIAL IMPACT

**FIGURE 9:**
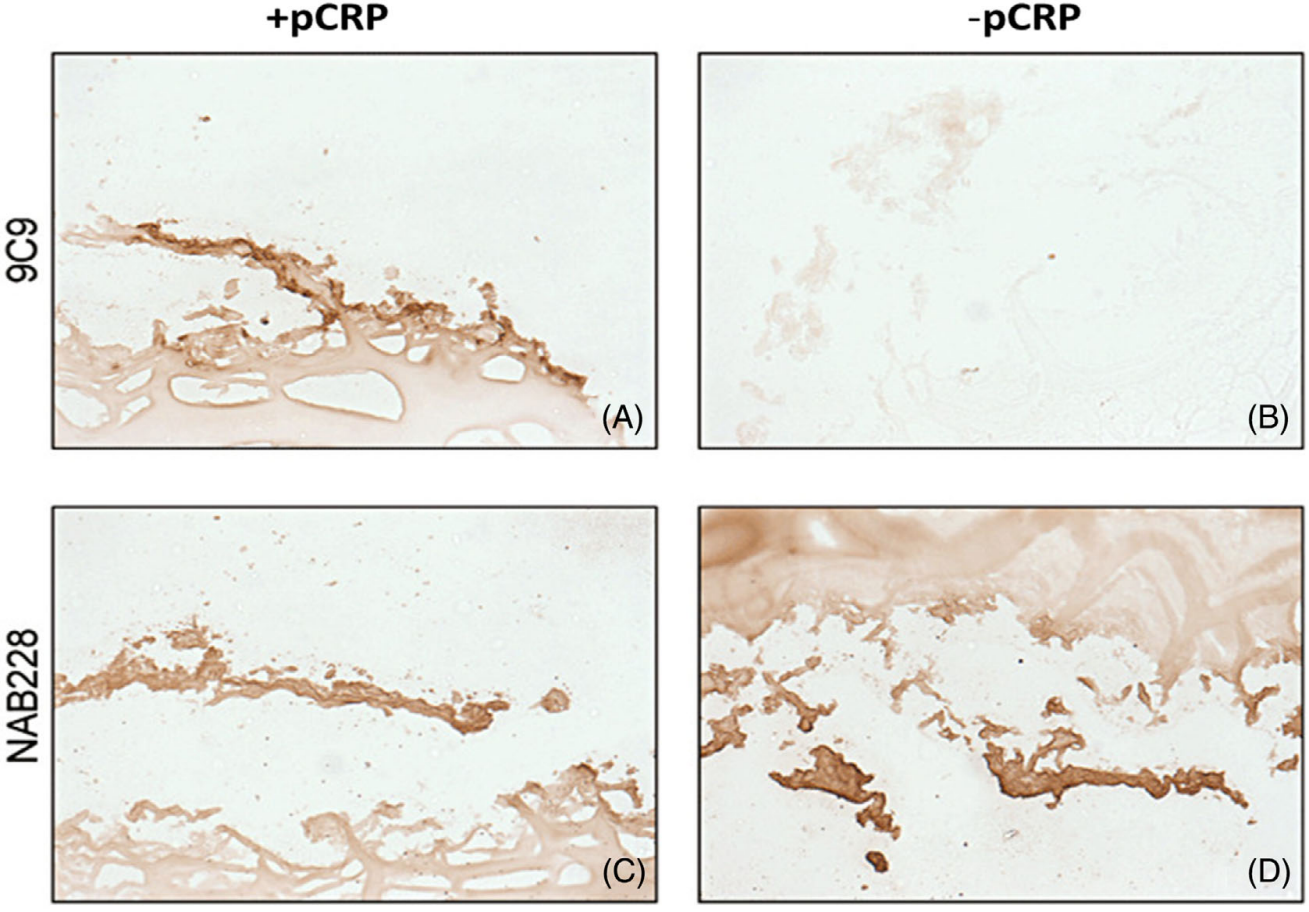
An artificial Aβ plaque incubated with pCRP (A) and fixed in agarose showing positive (DAB‐brown) staining with antibody clones 9C9 (mCRP) and NAB228 (Aβ) and also weakly for antibody 8D8 recognizing pCRP, however, plaques incubated without pCRP (B–D), fixed and stained showed no staining for 8D8 or 9C9 (B). Positive staining (brown) is seen with NAB228 (D). Taken from Strang et al. [52] with permission of the publisher.

In the normal human brain, there is a weak expression of mCRP in the cytoplasm of normal neurons [45]. As early as 1997, Duong et al. [51], identified increased expression of CRP (since mCRP was not realized at this time), immunoreactivity within both plaques and neurofibrillary tangles (NFTs) of post‐mortem specimens from individuals who died with AD. Later, Strang et al [52], showed that mCRP positive IHS staining was associated with AD‐positive regions in post‐mortem cortical brain specimens, and that incubation of pCRP with Aβ(42)‐peptide in vitro, led to the generation of mCRP‐specific inflammatory Aβ‐plaques (Figure 9), whilst cerebral expression of CRP correlated with Aβ1‐42 and serum amyloid P component (SAP) indicating neurodegenerative capacity in a transgenic murine amyloid precursor protein (APP)/presenilin‐1 (PS1) model [53].

During both acute and chronic inflammation, circulating soluble nCRP is produced primarily by the liver, however, the sensor for continued synthesis and secretion is conversion rate to mCRP at sites of cellular and tissue damage. The half‐life of nCRP is around 19 h, and nCRP does not “home” to sites of tissue injury, whilst mCRP does, it can be effectively proteolyzed by neutrophil‐derived peptidases. Therefore, mCRP identified within the brain, and appearing to traverse through the micro‐circulation, being identifiable even in the hypothalamus, suggests an endothelial‐activation‐specific process in addition to somehow a reduced capability within damaged brain vasculature for effective clearance of deposited mCRP [54].

**FIGURE 10:**
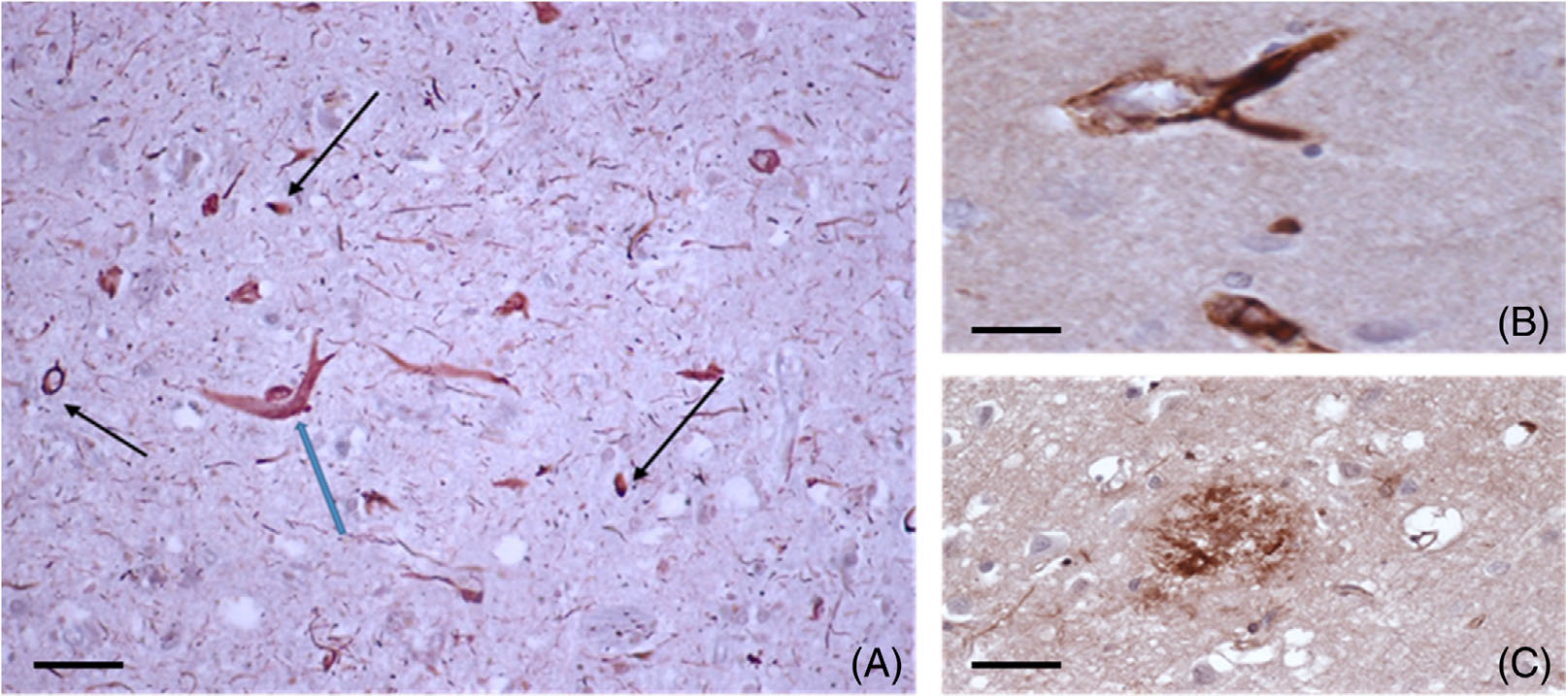
Chronic extra‐cellular expression of mCRP in the cortical tissue stroked AD brain, from 12. (A) Showing strong staining in neuritic plaques (blue arrow), fibril‐like structures, and immune infiltrating cells (DAB‐brown; mCRP; gray‐black‐p‐Tau double‐labelled). (B) is a magnified image of a neuritic plaque intensely “coated” with mCRP and (C), mCRP positive region with the appearance of a cortical plaque (DAB brown single stain), (A; Scale bar in [A] 100 μm and [B,C] 200 μm). Post‐mortem samples were obtained from ischemic stroke victims with AD: Samples were obtained from the Institute of Neuropathology Brain Bank, University Hospital of Bellvitge and the images are reproduced with agreement of the publisher.

Considering the topological localization of mCRP described in neuronal peri‐nuclear, nuclear, and axonal regions, and the emerging role of neuronal pentraxin receptors (probable binding partners of mCRP) in neurodegeneration and their combined role in mediating synaptic regulation and dysfunction [55], the impact upon signaling, NVU patency and BBB breakdown should be investigated in greater detail (Figure 10).

It is important to be certain of the total antibody specificity, and in particular, the capability to discriminate mCRP from nCRP. In this regard, the mCRP‐binding antibodies that have been used in all the figures above within this review, have been previously and fully characterized by Potempa et al. and other groups, and are considered fully and completely specific. Appropriate controls were used in all studies where the native/pentameric nCRP antibody staining was compared in serial sections to ensure processing artefacts were not being observed. In all studies, a mild antigen retrieval protocol involving only citrate buffer was used and this has been shown in many previous studies not to affect the epitope conformation. In addition, earlier studies compared frozen sections without processing and showed similar expression profiles for mCRP. Intracellular staining can only be that of mCRP based upon the known properties and solubility of this protein and similarly luminal vascular expression represents binding with endothelial intima regions as shown in many previous cardiovascular‐themed studies. Therefore, we are confident in the accuracy of the experimental IHC data provided above.

## 6 MCRP AND THE NEUROVASCULAR UNIT

Neurovascular unit dysfunction with glial involvement, is now considered an early event in the development of dementia, with vascular amyloid beta deposits reducing blood flow, clearance capacity, BBB integrity, and subsequent induction of neuroinflammation [56]. Recently, Huang et al. [57], defined microglia dysfunction and its critical role in mediating pericyte loss and abnormal vessel contraction concomitant with NVU and BBB breakdown. Loss of white matter capillary pericytes were associated with ischemic stroke changes and all types of dementia with BBB dysfunction [58], and the impact upon reduction in capillary blood flow, IPAD and Aβ clearance eloquently described by Fisher et al [59]. Perivascular damage in TBI has also been clearly characterized with a critical neuroinflammatory buildup of phosphorylated tau protein concomitant with increased CRP, ICAM‐1/VCAM‐1 and BBB leakage in the microvasculature of post‐mortem samples of frontal cortex [60]. Emerging evidence suggests that deposition of mCRP may be an early pathological indicator of, and indeed a cause of NVU damage directly linking neuroinflammation of any cause with risk of dementia and synucleinopathies et al. and should be investigated in more detail [61].

## 7 DE NOVO SYNTHESIS AND SECRETION OF MCRP IN NEURODEGENERATION?

Evidence from the last two decades demonstrates that neurons possess the capability of de novo intracellular synthesis of mCRP and its upregulation within pyramidal neurons correlated with amyloid P expression and AD [62]. One question is, what purpose would neurons have for synthesizing their own mCRP intracellularly? The ultimate fate of the synthesized neuronal mCRP may be intracellular, possibly associated with the cytoskeleton and intermediate filaments or, alternatively (or as well as), secreted into the local environment of the neurons where it could contribute to the fibrous connective tissues and be an important component of cell–cell interactions within the neuronal environment, even for example, modulating the hyaluronic acid‐based ECM micro‐environmental stability and plasticity [63].

Since morphologically mCRP appears to be associated with intermediate filaments and can potentially self‐polymerize as it can directly promote the formation of tau fibrils in vitro; [12], it may act as a conduit from the nucleus of one cell (through laminins and the nuclear membrane) through the cytoplasm, through the plasma membrane, and into the extracellular space. Here, the extracellular mCRP may contribute to the matrix enabling a continuation of the fibrous network, although, currently, this is only a hypothesis. Thus, through the interactions of mCRP, the nucleus of one cell could directly “communicate” with the nucleus of a juxtaposed cell. This would allow coordination of function such as contact inhibition of growth that prevents tumor formation; [64], and hence the possible role of mCRP in co‐ordination of nerve transmissions should be studied. The most likely passage mechanism in and out of the cells is across the plasma membrane involving cholesterol, which is evenly distributed on the inside and outside leaflets, and involves the cholesterol binding site through residues 35–47, as described earlier. However, the role of mCRP either inside, or via membrane receptor‐associated signaling from outside the neuronal cells is not entirely understood [65]. Other short pentraxins form this highly conserved group, such as SAP, which is strongly associated with amyloid deposition, whilst neural pentraxin‐1 is associated with synaptic plasticity, however, its over‐expression in AD is linked to loss of synaptic connections and increased tau build‐up in dystrophic neurites, with a potential link to C1q‐mediated synaptic pruning and therefore mCRP pentraxin effects, providing further evidence of the importance of the “pentraxin family” in coordinating neural activity [66, 67].

**FIGURE 11:**
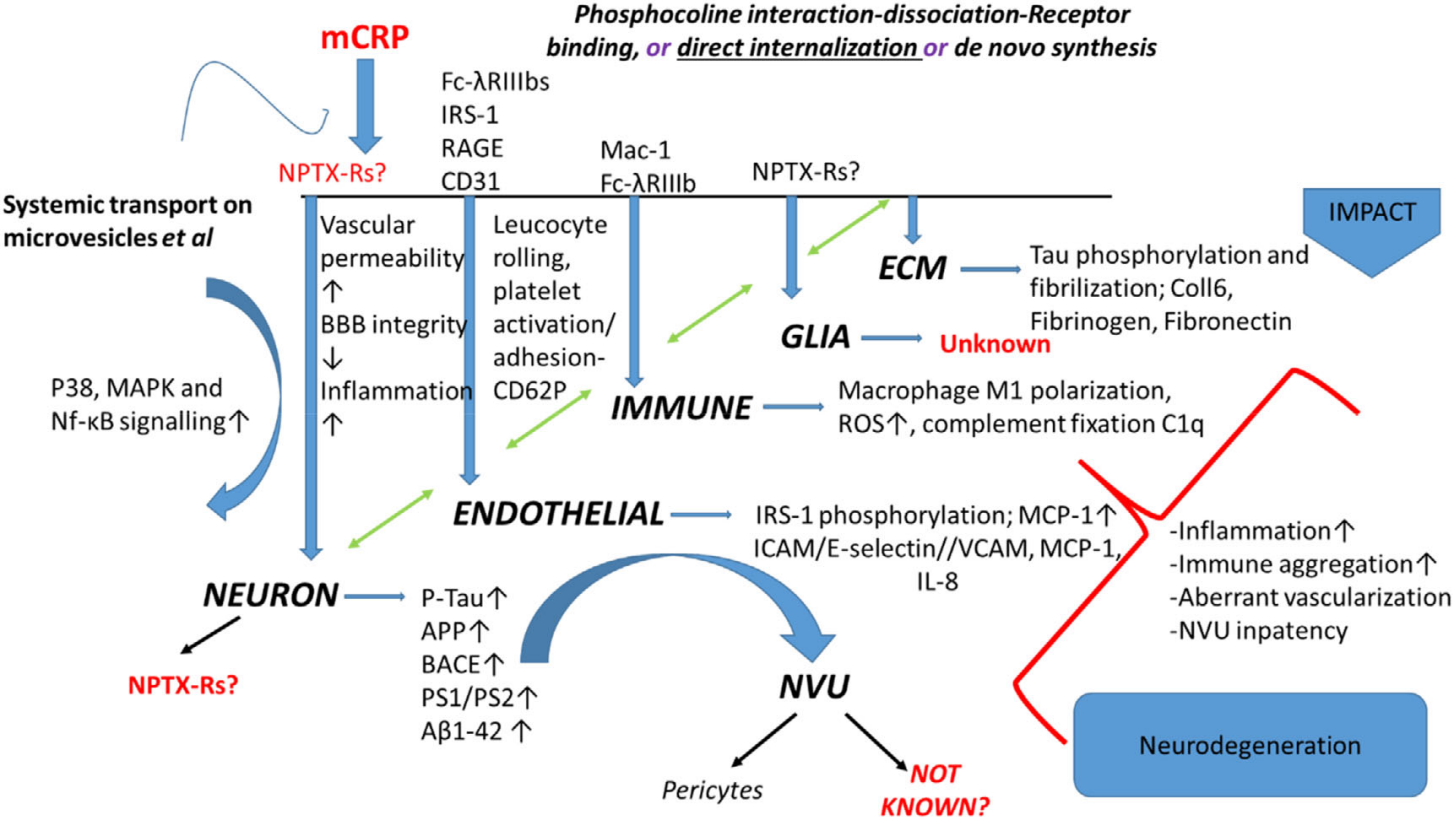
Monomeric C‐reactive protein (mCRP) can undergo direct internalization without receptor binding in endothelial cells (ECs) [68] and via Fc‐λRIIIb increased ICAM‐1/VCAM‐1 expression and MCP‐1/IL‐8 secretion in human coronary artery ECs [72]. mCRP‐induced phosphorylation of insulin receptor substrate‐1 (IRS‐1) in human brain microvessel EC in vitro [12], CD31, in APOE4 knock‐in mice associated with T‐lymphocyte extravasation and stunted vasculature following intra‐peritoneal injection [43], and binding and activation of the RAGE receptor [73]. Fc‐λR also mediates immune cell (macrophage/neutrophil) binding of mCRP via Fc‐λRIIIb, resulting in increased cellular activation. Limited mostly to the work of Bi et al. [53], mCRP has been shown to promote expression of the majority of AD/neurodegenerative proteins including APP, beta‐site amyloid precursor protein cleaving enzyme (BACE), PS1‐2, and Aβ1‐42, as well as Tau phosphorylation, but the neuronal‐mCRP receptors (one possibility being neuronal pentraxin receptors [NPTX‐Rs] are still not characterized [12, 74]). Within the ECM, mCRP recruits C1q binding and complement activation with combined deposition in damaged tissue; mCRP also induces platelet binding to collagen VI and binds directly to fibronectin, these interactions leading to vascular build up and damage as well as macrophage M1 polarization [71]. Interactions with receptors of glia and astrocytes are not reported in the literature.

Additional mCRP could be derived following synthesis and secretion, from activated ECs, M1‐polarised macrophages and glia, as reviewed by Sproston and Ashworth [5, 68] and showed within inflammatory hemorrhagic regions of brain following stroke as previously described [44]. For example, in EC, almost two decades ago, Venugopal et al. [69], showed “CRP” generation by human aortic ECs exposed to Il‐1/Il‐6 or TNF‐α, in vitro, whilst the macrophage cell line U937 replicated this effect in the presence of lipopolysaccharide (LPS) [70]. The overall contribution of this de novo synthesis remains controversial and requires further characterization (Figure 11) [71].

## 8 THERAPEUTIC IMPLICATIONS

**FIGURE 12:**
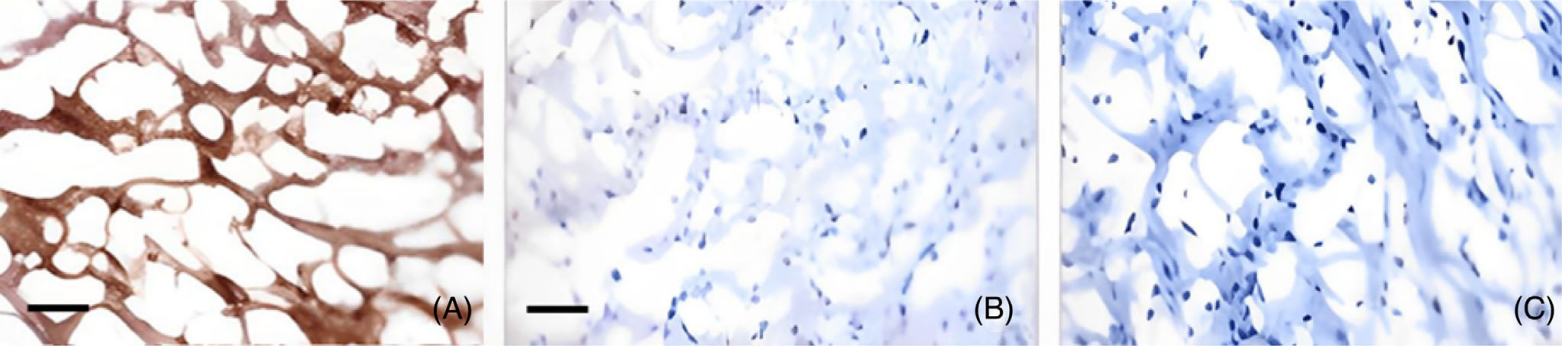
Human monomeric C‐reactive protein (mCRP) but not pCRP was detected in infarcted rat myocardium after administration of human pCRP into the circulation (A). Non‐infarcted right ventricular tissue from the same animal was used as a control and showed no evidence of mCRP or pCRP deposition (C). When pCRP was pre‐incubated with 1,6‐bis‐PC (50:1 molar ratio), there was no significant deposition of mCRP or pCRP in either the infarcted (C) or non‐infarcted tissue. Figure used with the permission of the journal [6]. Scale bar in (A) 100 μm.

The published work to date indicates a powerful pathological role of mCRP in many of the processes that can both initiate, and perpetuate, neuro‐inflammatory‐associated vascular and neurovascular damage resulting/contributing to development of potentially all types of dementia. It is pertinent to ask the question, could an inhibitor of mCRP binding, or a pCRP dissociation blocker present as a useful “vascular protection” therapeutic? In this regard, Thiele et al [6, 75] described production of a synthetic binding molecule‐1,6‐bis(phosphocholine)‐hexane (1,6‐bis‐PC) which stabilized the pentameric molecule of CRP preventing phospholipase A2 (PLA‐2)‐mediated LPC generation on the surface of “membranes” and subsequent LPS‐dependent pCRP‐dissociation. The inhibitor successfully reduced LPS‐induced PLA‐2 activity in human mononuclear lymphocytes and furthermore, blocked mCRP deposition in infarcted myocardial tissue of rats (Figure 12), concomitantly reducing local inflammation.

**FIGURE 13:**
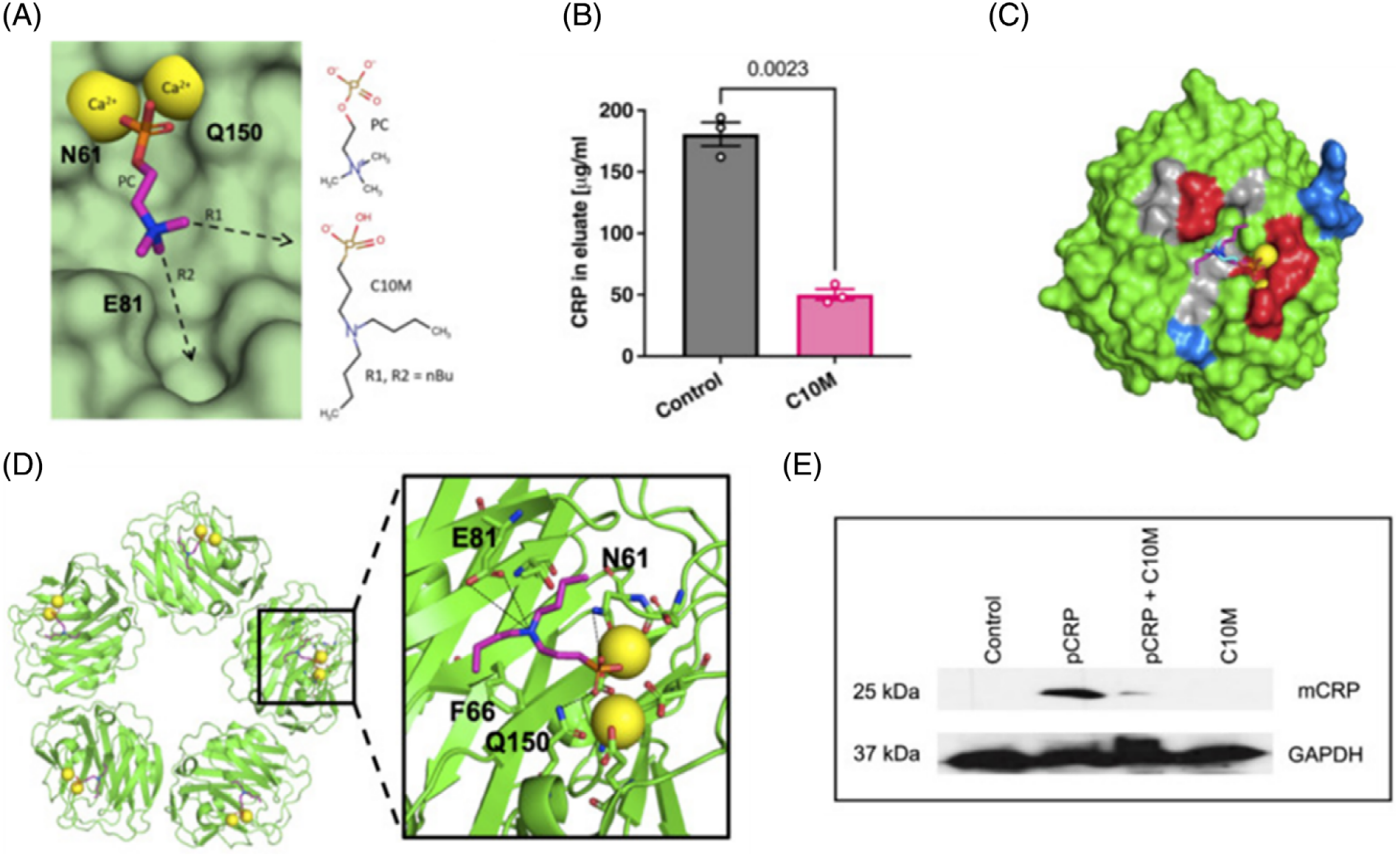
(A) Shows design of the phosphonate compound C10M based on binding characteristics of PC:pCRP and two n‐butyl substituents on the tertiary amine in the binding pocket via vectors R1 and R2. Calcium cations: yellow spheres and pCRP residues Asn 61 (N61), Glu 81 (E81), and Gln 150 (Q150) are indicated. pCRP; light green. (B) C10M reduced binding of pCRP to immobilized PC. pCRP with p‐aminophenyl phosphoryl choline agarose beads under porous solid column chromatography was used to evaluate the binding capacity of pCRP to PC. (C) Indicates the crystal structure of pCRP complexed with C10M (red spheres) integral to the pocket as PC/PE. Pentamers 2 and 4 (gray) show relative location to pentamers 1 and 3 (green). (D). One mCRP unit contained two calcium cations (yellow spheres). Interaction of the phosphonate moiety with the bound calcium cations (yellow spheres) and hydrogen bonds anchor C10M to the binding pocket. Alignment of C10M (pink/blue/red/orange sticks) and PC (cyan/blue/red/orange sticks) in complex with pCRP via the Cα atoms of pCRP. One monomeric subunit of pCRP is shown as the “surface”, the location of acidic (red), basic (blue) and hydrophobic (gray) residues around the PC binding pocket is indicated. (E); Western blot of pCRP binding to activated platelets. Platelets incubated with C10M could not effectively bind with CRP (using anti‐CRP antibody) and GAPDH acts as the control. Permission for the use of the figure was granted by the journal.

Recently, Zeller et al. [76], using x‐ray crystallography and affinity chromatography, developed a new compound—(3‐{dibutylamino}propyl) phosphonic acid (C10M) that, as a small molecule inhibitor, was able to block the PC interaction with pCRP and prevent binding and dissociation as well as CRP‐platelet interactions (Figure 13).

The PC binding pockets are also susceptible to other structurally similar orphan drugs including acetylcholine and nicotine, and effectively blocked monocyte mCRP‐induced, U937‐associated EC adhesion, pro‐inflammatory cytokine expression and cell signaling, as well as platelet aggregation [77]. Whether these SMIs could reach constant therapeutically active levels beyond the BBB, protect the brain vasculature and enhance NVU stability in acute inflammatory pathologies such as stroke, and/ or chronic inflammation as in auto‐inflammatory disease remains unknown; however, an additional limitation might be that mCRP could start to be “laid down” in brain tissue early in the disease process and would seem to remain stably (as morphological and histological studies have shown) with no known mechanism to remove. A number of animal “models” could also be utilized in order to demonstrate more detailed cellular or NVU based interactions with mCRP in vivo. For example, a murine model of vascular disease could be particularly useful since CRP is not associated with either complement activation or inflammation and hence, can be completely artificially induced to represent more accurately human neurodegenerative pathology. In addition, larger more representative models, such as canine dementia or AD, could be utilized to both predict and also follow in stages the pre‐symptomatic changes and the relative involvement of mCRP [78].

In any case, protection of vulnerable individuals by risk stratification or clinical history and reduction of subsequent risk of acute vascular perturbation might be possible as we learn more about the mechanisms of cellular activation along with conduction of proof‐of‐concept studies.

## AUTHOR CONTRIBUTIONS

Ylenia Pastorello, Mark Slevin, Roxana O. Carare, Mario Di Napoli, Claudia Banescu, and Lawrence Potempa all contributed to the drafting of the manuscript.

## CONFLICT OF INTEREST STATEMENT

The authors declare no conflicts of interest.

## FUNDING STATEMENT

Noi inhibitori ai proteinei C‐reactive și blocanți de disociere pentru uz terapeutic în bolile cardiovasculare–CREDICARD, Contractului de finanțare nr. PCE 60/2021, cod proiect: PN‐III‐P4‐ID‐PCE‐2020‐1540.

## Data Availability

All data is freely available to share from the Brain Pathology web‐site.
